# A Combined Molecular Dynamics and Hydropathic INTeraction (HINT) Approach to Investigate Protein Flexibility: The PPARγ Case Study

**DOI:** 10.3390/molecules29102234

**Published:** 2024-05-10

**Authors:** Federica Agosta, Pietro Cozzini

**Affiliations:** Molecular Modelling Lab, Food and Drug Department, University of Parma, Parco Area delle Scienze, 17/A, 43121 Parma, Italy; federica.agosta@unipr.it

**Keywords:** Molecular Dynamics, HINT force field, PPARγ, conformational analysis, mechanism of action

## Abstract

Molecular Dynamics (MD) is a computational technique widely used to evaluate a molecular system’s thermodynamic properties and conformational behavior over time. In particular, the energy analysis of a protein conformation ensemble produced though MD simulations plays a crucial role in explaining the relationship between protein dynamics and its mechanism of action. In this research work, the HINT (Hydropathic INTeractions) LogP-based scoring function was first used to handle MD trajectories and investigate the molecular basis behind the intricate PPARγ mechanism of activation. The Peroxisome Proliferator-Activated Receptor γ (PPARγ) is an emblematic example of a highly flexible protein due to the extended ω-loop delimiting the active site, and it is responsible for the receptor’s ability to bind chemically different compounds. In this work, we focused on the PPARγ complex with Rosiglitazone, a common anti-diabetic compound and analyzed the molecular basis of the flexible ω-loop stabilization effect produced by the Oleic Acid co-binding. The HINT-based analysis of the produced MD trajectories allowed us to account for all of the energetic contributions involved in interconverting between conformational states and describe the intramolecular interactions between the flexible ω-loop and the helix H3 triggered by the allosteric binding mechanism.

## 1. Introduction

The rapid and exponential development of computer hardware, software, and algorithms has increased molecular modelling applications in chemistry and life science.

In particular, molecular modelling has proven to be a valid support to medicinal chemists and pharmacologists in guiding the cost-effective identification of biological targets and active compounds, reducing the number of experimental tests, and limiting the use of animal models [1,2,3].

Among computational techniques, Molecular Dynamics (MD) aims to evaluate a molecular system’s thermodynamic properties and conformational behavior over time [4,5]. Although the advent of High-Performance Computing (HPC) has enabled increased simulation timescales and broadened the applications of Molecular Dynamics [6,7,8], evaluating the thermodynamic stability of intrinsically disordered proteins or flexible biological targets requires an accurate analysis of produced MD trajectories to assess intramolecular stability and key interaction-pattern changes in determining conformational transitions [9].

The more flexible the protein is, the greater the number of conformations it can adopt, and each conformation is separated from the others by an energy barrier and a different pattern of intramolecular interactions.

The energy analysis of a system’s conformations ensemble plays a crucial role in deciphering the relationship between protein dynamics and its mechanism of action and requires the application of sensitive and accurate energy scoring functions.

HINT, Hydropathic INTeractions, is a LogP_o/w_-based scoring function conceived for quantifying hydrophobic and polar interactions between or within molecules in a biological environment [10,11] in terms of a score summing atom–atom interactions. The HINT output file records and accounts for favorable (hydrogen bond, electrostatic contributions, and hydrophobic interactions) and unfavorable (desolvation energy and repulsive Coulombic interactions) interactions between noncovalently bonded atom pairs.

Moreover, as the LogP_o/w_ (partition coefficient for solute transfer in 1-octanol/water) is a thermodynamic parameter that implicitly also includes the hydrophobic contribution generated by the desolvation process involving interacting molecules (entropic contribution), the HINT score is directly related to the system free energy [12,13].

Recently, we used HINT to evaluate the stability of a protein in response to single and multiple site mutations and different pH conditions, demonstrating how a HINT-based analysis of different protein conformations reveals specific information on the energetic contributions involved in interconverting between conformational states and allows for a detailed characterization of the more stable and populated state [14,15].

In this research work, for the first time, HINT was used to handle a Molecular Dynamics trajectory with the aim to evaluate the thermodynamic stability of a biological system during all the simulation times. In particular, the HINT-based analysis was employed to investigate the PPARγ mechanism of activation.

The Peroxisome Proliferator-Activated Receptor γ (PPARγ) is a nuclear receptor acting as a ligand-dependent transcription factor involved in glucose and lipid metabolism [16,17]. Although PPARγ shares the identical 12-helix structural arrangement with other nuclear receptors, it is also characterized by a flexible ω-loop (residues 265–276) responsible for increased active site flexibility and ligand binding mechanism complexity. Its active site’s higher volume and flexibility are related to the ability to bind more than one ligand simultaneously [18].

Given the PPARγ central role in various types of physiological and pathological events, including anti-inflammatory actions and carcinogenesis, understanding the molecular basis of its activation mechanism has become increasingly crucial for a proper rational design of potential drugs [19,20].

In this research work, we applied our computational pipeline, combining Molecular Dynamics and an intramolecular conformation ensemble analysis, to decipher the PPARγ mechanism of action at a molecular level. We focused on the PPARγ complex with Rosiglitazone, a synthetic anti-diabetic compound and investigated the Oleic Acid allosteric modulation effect on the receptor conformational stability.

## 2. Results and Discussion

### 2.1. Active Site Analysis

The PPARγ ligand binding domain (LBD) secondary structure consists of 12 α-helices and four stranded β-sheets. It is characterized by a large and flexible active site with a particular Y-shape able to bind structurally different natural and synthetic ligands [18]. The C-terminal end of the LBD includes the activation function domain (AF-2) helix H12, which, after ligand interaction, reduces receptor flexibility and promotes co-activator binding, essential for PPARs’ transcriptional activity [21].

Fatty acids and eicosanoids have been identified as PPARs natural ligands [22], while glitazones, such as Rosiglitazone, have been developed as PPARγ synthetic agonists conceived for the treatment of type II diabetes [23,24].

An F-pocket analysis of the PPARγ–Rosiglitazone complex (PDB ID: 1FM6, chain D) underlines the active site’s high volume (1413.471 Å^3^) and flexibility (Table 1), when compared to other nuclear receptors (PPARγ and Estrogen Receptor (Erα) comparison in Supplementary Table S1). The binding site presents a prevalent hydrophobic character justifying the PPARγ ability to bind fatty acids and hydrophobic natural and synthetic compounds.

**Figure 1:**
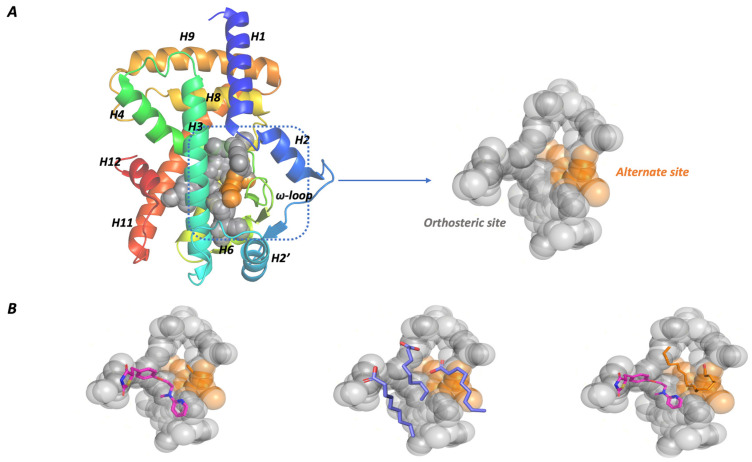
PPARγ active site characteristics. (**A**) PPARγ active site presents a high volume and is defined between helix3, helix12, and the flexible ω-loop. It can be divided into two regions: the orthosteric site (grey) and the alternate site (orange). (**B**) PPARγ presents different activation mechanisms. A single compound can bind the orthosteric site with a consequent canonical activation mechanism. The active site high volume allows for the interaction of two or more ligands simultaneously. One of the co-bound ligands is located in the alternate site (PDB ID: 1FM6, 4EM9, and 6MD4).

The PPARγ binding pocket can be divided into two regions: the orthosteric (or canonical) binding site and the alternate binding site [25], as shown in Figure 1A. The first includes residues in helices 3, 5, 6, and 7 and the b-sheets and presents a peculiar Y-shape consisting of Arm I and Arm II regions.

Arm I includes polar residues such as Ser289, Tyr327, His449, and Tyr473, which are involved in hydrogen bond interactions with the carboxylate group of fatty acids, while Arm II presents a prevalent hydrophobic character.

The alternate site is a solvent-accessible area between helix H3 and the ω-loop.

This characteristic active site structure is responsible for different PPARγ activation mechanisms (Figure 1B).

Different experimental assays, such as time-resolved fluorescence resonance energy transfer (TR-FRET) [25], NMR spectroscopy, and X-ray determination [26], revealed the cooperative co-binding effect of multiple ligands to PPARγ. The ligand interaction with the orthosteric site induces a canonical activation mechanism involving the AF-2 motif conformational change and the co-activators’ recruitment. An alternative activation mechanism is possible when two or three molar equivalents of the same ligand or two different ligands interact with PPARγ simultaneously, and one molecule is located in the alternate site, where it acts as an allosteric modulator, affecting the pharmacological effect of the orthosteric ligand.

### 2.2. PPARγ Ligand Binding Mode

**Figure 2:**
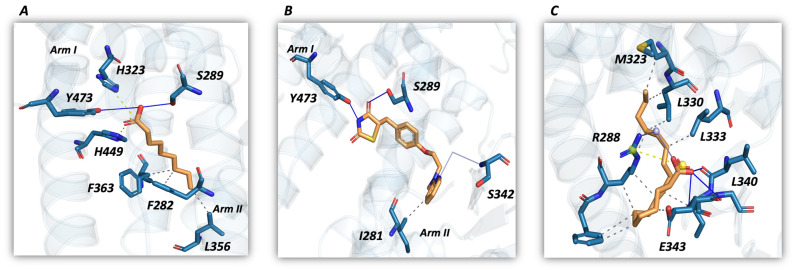
PPARγ ligand binding mode. Ligand interactions are shown by employing the PLIP plugin in Pymol. Protein is shown in the transparent cartoon, ligand as orange stick, and interaction types are depicted according to PLIP representation: hydrogen bonds as blue lines, salt bridge as dotted yellow lines, and hydrophobic interactions as dotted grey lines. (**A**) Fatty acids are PPARγ natural ligands (PDB ID: 4EM9) that are able to interact with the Orthosteric binding site through salt bridges with histidine residues and hydrogen bonds with residues in Arm I, while the hydrophobic tail is stabilized through van der Waals interactions. (**B**) Rosiglitazone (PDB ID: 1FM6) is one of the thiazolidinediones used for type II diabetes treatment and shares the same interaction pathway with fatty acids. (**C**) Fatty acids can also interact with the alternate site when another ligand is bound to the Orthosteric one (PDB ID: 6MD4). This interaction involves a salt bridge with Arg288 and hydrophobic interactions.

Fatty acids are PPARγ natural ligands. Arm I polar residues interact with the carboxylate group: His323 and His449 are involved in salt-bridges interactions, while Ser289 and Tyr473 stabilize the ligand through hydrogen bonds. Arm II residues stabilize the hydrophobic fatty acids tail via van der Waals interactions (Figure 2A) [27,28].

Glitazones like Rosiglitazone are synthetic anti-diabetic compounds with the same interaction pathway described for natural ligands (Figure 2B). The thiazolidinedione group is stabilized via hydrogen bond interactions with Arm I polar residues, while the hydrophobic scaffold is located in the Arm II region (Figure 2B).

Ligand interactions with orthosteric site polar residues produce an AF-2 stabilization effect with a consequent transcription activation effect. This binding mode is related to glitazones’ anti-diabetic efficacy but is also responsible for different side effects, such as water retention and bone loss [29].

The alternate site presents a prevalent hydrophobic character, except for Arg288 and Glu343, two charged residues involved in electrostatic and polar interactions (Figure 2C). Ligand interaction in the alternate site can synergize or allosterically modulate the orthosteric ligand efficacy.

The glitazones and fatty acids’ synergic interaction with PPARγ has been related to an increased anti-diabetic efficacy thanks to the stabilization of the region near the ω-loop that has been associated with a phosphorylation inhibition of S245 mediated by the Cdk5 kinase [30,31,32,33].

### 2.3. Molecular Dynamics

To investigate the PPARγ mechanism of activation, we analyzed the stability of PPARγ in complex with Rosiglitazone and both Rosiglitazone and Oleic Acid through Molecular Dynamics. All simulations were carried out in triplicate for 250 ns.

**Figure 3:**
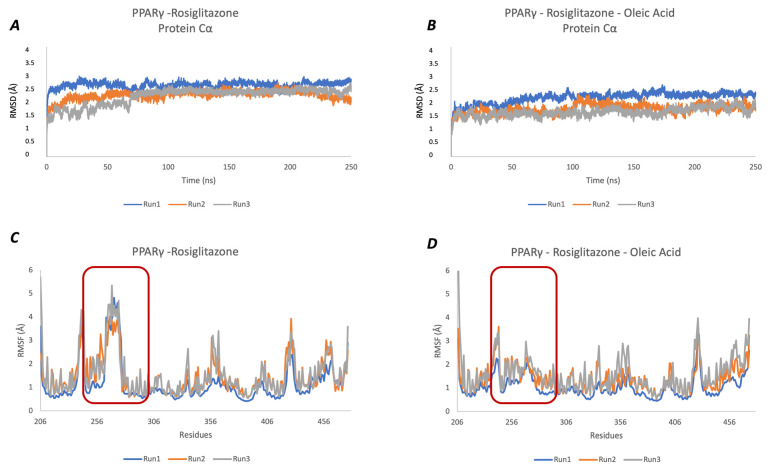
MD analysis. (**A**) The RMSD analysis of the PPARγ–Rosiglitazone complex (PDB ID: 1FM6) shows the high stability of the system during all simulation times in all three replicas (Run1 in blue, Run2 in orange, and Run3 in grey). (**B**) The RMSD profile of the PPARγ–Rosiglitazone–Oleic Acid complex (PDB ID:6MD4) in three independent replicas reveals system stability (Run1 in blue, Run2 in orange and Run3 in grey). The protein RMSF profile is shown in (**C**) (PPARγ–Rosiglitazone system) and (**D**) (PPARγ–Rosiglitazone–Oleic Acid complex). The red rectangle highlights the flexible regions corresponding to the ω-loop residues (265–276 residues). A second ligand bound to the alternate site produces a significant ω-loop stabilization.

As shown in Figure 3A,B and in Supplementary Table S2, both systems are stable during the simulation time in all three produced trajectories.

The protein RMSF profile (Figure 3C,D) reveals higher flexibility of residues belonging to the ω-loop (residues 265–276 in red rectangle) with a significant difference between the two protein systems. The ω-loop is the structural element responsible for active site flexibility that plays a crucial role in the AF-2 motif structural conformational change that is essential for co-activators’ recruitment and transcription activation.

The Oleic Acid bound to the alternate site reduces the ω-loop flexibility, as shown in Figure 3D.

In the PPARγ–Rosiglitazone complex (Figure 3C), the ω-loop RMSF ranges between 4 and 6 Å, while the Oleic Acid co-binding produces a significant stabilization, with RMSF values between 2 and 3 Å (Figure 3D).

Ligands remain stably bound to the orthosteric and alternate site, respectively (ligand RMSD profile in Supplementary Figure S1 and Table S3).

**Figure 4:**
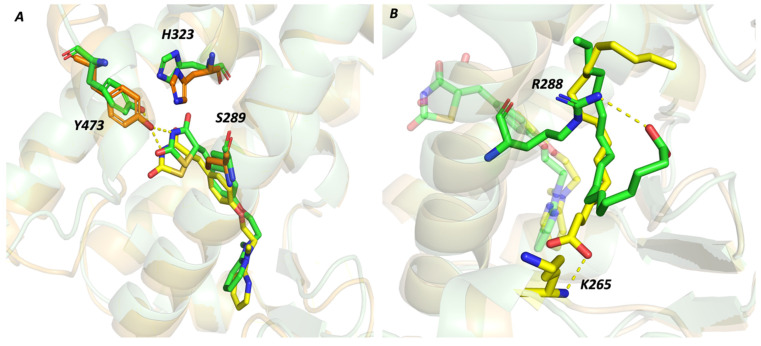
Rosiglitazone and Oleic Acid interactions during the simulation time. Rosiglitazone (**A**) is stable in both system thanks to h-bond interactions with polar residues (His323, Ser289, and Tyr473). The Oleic Acid (**B**) adopts two different conformations responsible for its higher RMSD value. These conformations differ for the orientation of the acidic group that can be stabilized through salt bridge interactions with Arg288 (green conformation) or with Lys265 (yellow conformation).

In particular, Rosiglitazone remains anchored to the orthosteric site thanks to a network of hydrogen bonds involving the thiazolidine-2,4-dione group and the Ser289, Tyr473, and His323 residues in polar Arm I (Figure 4A).

Although stable, Oleic Acid explores two different conformations in the alternate site that might justify its higher RMSD value when compared to Rosiglitazone (Supplementary Table S3). Ligand conformations differ according to the orientation of the acidic group that can be stabilized through a salt bridge interaction with Arg288 or can be oriented toward the Lys265 (Figure 4B). In both conformations, the ligand scaffold is stabilized through hydrophobic interactions with residues belonging to the hydrophobic alternate site (Leu228, Ala292, Leu333, and Met329).

However, Oleic Acid is not directly involved in intermolecular interactions with ω-loop residues, whose reduced flexibility could be attributable to a more complex protein conformational arrangement.

A detailed analysis of the intramolecular stability of the two protein systems over time was performed in HINT.

### 2.4. HINT Based Analysis

The Intramolecular HINT (Hydropathic INTeractions) energy scoring function is a fast and reliable tool that is able to estimate minor energy differences in the intramolecular interaction pattern and evaluate protein thermodynamic stability [11]. HINT uses experimental LogP_o/w_ values, providing an overall representation of the energy profile, considering both enthalpic and entropic contributions to the ΔG [10]. This force field has been successfully employed for the analysis and comprehension of various biological problems, ranging from protein–ligand [34] to protein-protein [13] and protein–DNA interaction energy evaluations [35]. In this research work, the HINT force field was used to analyze the molecular basis of the ω-loop stabilization effect produced by the Oleic Acid bound to the alternate site. The HINT output file provides a detailed description of each energy contribution (hydrogen bond, electrostatic, and hydrophobic interactions) occurring between all atom pairs, allowing for a clear representation of different protein conformations and an in-depth analysis of the network of intramolecular interactions.

**Figure 5:**
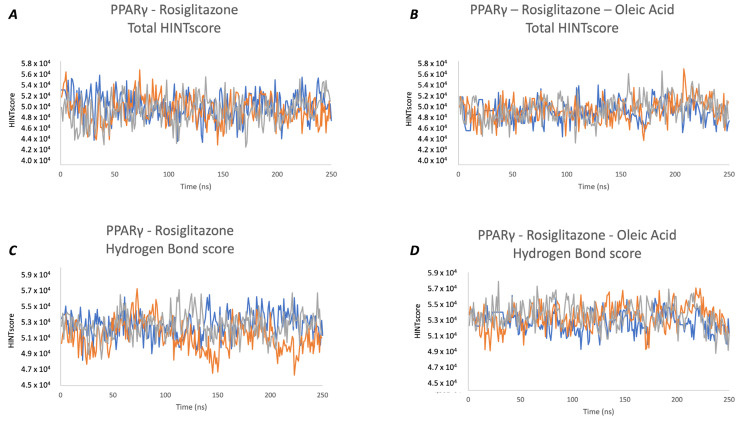
HINT profiles. PPARγ–Rosiglitazone (**A**) and PPARγ–Rosiglitazone–Oleic Acid complex (**B**) presents a comparable average total HINT score in all three produced trajectories (Run1, Run2, and Run3 are represented as blue, orange, and grey lines, respectively). Although stable, systems are significantly different for h-bond energy contribution to the total HINT score (PPARγ–Rosiglitazone (**C**) and PPARγ–Rosiglitazone–Oleic Acid complex (**D**). This energy contribution is greater in the PPARγ–Rosiglitazone–Oleic Acid complex, suggesting a different intramolecular connection pattern.

The intramolecular HINT energy profile reveals that both analyzed systems are equally stable during all simulation times, with a comparable average total HINT score in all three produced trajectories (as shown Figure 5A,B for PPARγ–Rosiglitazone and PPARγ–Rosiglitazone–Oleic Acid, respectively). All contributions to the total HINT score are described in detail in Supplementary Figure S2.

A deeper analysis of individual energy contributions to the total HINT score (hydrogen bond and electrostatic and hydrophobic energy contributions) highlights a different intramolecular energy stability related to hydrogen bond contributions to the total HINT score, suggesting a greater intramolecular stability of the PPARγ–Rosiglitazone–Oleic Acid complex and a different intramolecular interaction pattern (Figure 5C,D and Table 2).

The visual inspection of MD trajectories and the three-dimensional superposition of the extracted structures related to the lowest energy frames (higher total HINT score) unveils how the Oleic Acid bound to the alternate site triggers a protein conformational change.

**Figure 6:**
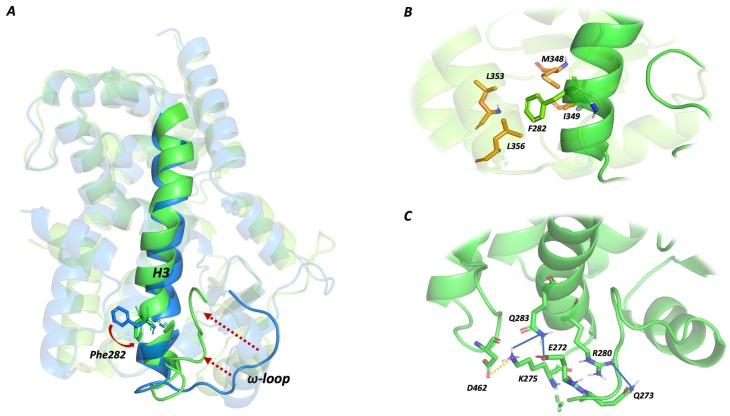
HINT profiles. Rosiglitazone–Oleic Acid co-binding effect. (**A**) PPARγ 3D structure alignment reveals a conformational variation of the ω-loop and helix H3. Based on HINT energy profile analysis, protein 3D structures were extracted from the MD trajectory. PPARγ–Rosiglitazone–Oleic acid complex is represented in green, while the PPARγ–Rosiglitazone complex is in blue. Structural changes are underlined through red arrows. (**B**) Protein structure reveals an alternative H3 conformation where the Phe282 phenyl ring (green sticks) is projected toward a hydrophobic task (orange residues). (**C**) ω-loop residues are stabilized thanks to an intensive intramolecular interaction pattern with H3 residues. Hydrogen bonds are shown as blue lines, while electrostatic interactions are shown as orange lines.

In this newly adopted conformation, the ω-loop is pushed toward the helix H3 (Figure 6A) and stabilized thanks to an intensive intramolecular interaction pattern involving residues 272–275 (Figure 6C).

In particular, the ω-loop is anchored to the helix H3 by a tight network of electrostatic and hydrogen-bond interactions involving Glu272, Gln273, and Lys275.

Gln273 interacts with Arg280 via a hydrogen bond, while Gln283 residue acts as bridging group, mediating a hydrogen bond network with Glu272 and Lys275. Lys275 is also involved in a salt bridge interaction with Asp462.

The conformation stabilization can also be ascribed to a three-dimensional arrangement of helix H3. The Phe282 phenyl ring is shifted toward hydrophobic residues such as Leu353, Leu356, Ile281, Met348, and Ile349, generating an intensive hydrophobic network and contributing to the overall stability.

Differences in intramolecular interactions between two or more protein conformations can be evaluated though a meticulous analysis of the HintTable output file.

As shown in Supplementary Figure S3, the HINT output file is a table containing all atom-by-atom interactions including parameters used for calculation (the hydrophobic atom constant, the SASA, and the distance between the interacting atoms). The final column lists the characterization of the interaction type (hydrophobic, acid-based, acid–acid, base–base, hydrophobic–polar, or hydrogen bonding). Positive values represent favorable interactions for intramolecular stability.

According to the HintTable output file, as described above, there is a significant difference in intramolecular interactions involving ω-loop residues between the two analyzed systems (PPARγ–Rosiglitazone complex in Supplementary Figure S3A; PPARγ–Rosiglitazone–Oleic Acid complex in Supplementary Figure S3B). Differences are related to residues 272–275, which, in the PPARγ–Rosiglitazone–Oleic Acid complex, are involved in an intensive interaction pattern with the helix H3 residue.

The overall stabilization of the region between the ω-loop and the helix H3 is related to a protein conformation less accessible to phosphorylation mediated by Cdk5. The presence of a second ligand bound to the alternate site would be related to a possible pharmacological synergic effect between the orthosteric ligand (Rosiglitazone) and the allosteric modulator (Oleic Acid), preventing or reducing post-transductional modification, such as phosphorylation, with a potential increase in anti-diabetic efficacy [36,37].

## 3. Materials and Methods

### 3.1. PDB Structure Analysis

PPARγ three-dimensional structures were retrieved from the Protein Data Bank [38] as follows: PPARγ–Rosiglitazone complex (1FM6, chain D) and PPARγ–Rosiglitazone-Oleic Acid complex (6MD4, chain A). The PPARγ–Rosiglitazone complex was used for the analysis of the active site in the F-pocket tool, using the default parameters setting [39].

Protein-Ligand 3D interactions were analyzed employing the Protein–Ligand Interaction Profile plugin (PLIP) implemented in Pymol [40,41].

### 3.2. Molecular Dynamics

Molecular dynamics simulations were performed in Gromacs (v.2021.4), choosing the Amber force-field (ff19SB) [42,43].

Structures were pre-processed in Sybyl v 8.1 (http://www.tripos.com/ accessed on 10 January 2008) to remove water molecules and co-factors. Hydrogen atoms were added and minimized using the Powell algorithm, with a coverage gradient of <0.5 Kcal (mol Å)^−1^ and a maximum of 1500 cycles.

Ligand parametrization was performed in Antechamber, choosing the General Amber Force Field (Gaff2) for atom-types assignment and AM1-BCC as the charge computing method [44,45].

Both systems were included in an octahedron box of 10 Å radius, solvated using the TIP3P water molecules model, and neutralized with NaCl, using a Monte-Carlo placing method and setting a salt concentration of 0.15 M.

Each system was minimized by restraining the backbone (k = 10 Kcal/mol A^−2^). The steepest descendent minimization algorithm was used during the initial cycles (1000 cycles), followed by the conjugate gradient method (maximum 5000).

Each minimized system was gradually heat from 0 to 300 K for 0.3 ns, followed by 3 ns of NPT simulation with a target temperature of 300 K.

During the NPT equilibration procedure, the temperature was maintained at 310.15 K using a Langevin thermostat (damping constant = 1 ps^−1^), and the pressure was maintained at 1 atm using a Berendsen barostat. Bond lengths involving hydrogen atoms were constrained using the M-SHAKE algorithm with an integration time step of 2 fs [46].

Long-range Coulomb interactions were handled using the Particle Mesh Ewald summation method (PME) [47]. A non-bonded cut-off distance of 9 Å was used.

Each system was simulated in triplicate for 250 ns, starting from different coordinates and velocities. The trajectory file was written every 20 ps.

The Root Mean Square Deviation (RMSD) and the Root Mean Square Fluctuations (RMSF) were calculated using Gromacs functions.

### 3.3. HINT Analysis

HINT (Hydropathic INTeractions) was used to evaluate the system stability during the MD simulation time and evaluate the effect of double-bound ligands on ω-loop and protein flexibility.

Protein partitioning, i.e., assigning atomic hydropathic parameters to each atom, was performed in HINT, using the “Dictionary” option and choosing the semi-essential hydrogen treatment that explicitly includes polar, unsaturated, and alpha to heteroatom hydrogens. Proteins were partitioned under a neutral pH condition [10].

The HINT score was calculated as a summation of hydropathic interactions between all atom pairs (∑∑*b_ij_*, *i* = 1 to N, *j* = *i* + 1 to N)—excluding those in 1–2 (bonded) and 1–3 (angle) sets—considering the hydrophobic atom constant (*a*), the solvent accessible surface area (SASA), and the functional distance behavior for the interaction (*R_ij_*):

$$B=\sum^{\;}\sum^{\;}b_{ij}$$

$$b_{ij}=S_{i\;}\;a_{i}\;S_{j}\;a_{j}\;R_{ij}$$

As the hydrogen atoms were modelled, hydrogen bonds were described considering heavy atom/heavy atom distances. Above 3.65 Å, the interaction was classified as acid/base.

HINT tab output files were analyzed to evaluate and compare the system’s stability. Positive values represent favorable interactions, such as hydrogen bond, acid–base, and hydrophobic interactions, while negative values represent unfavorable interactions, such as desolvation and repulsive Coulombic interactions.

## 4. Conclusions

Molecular Dynamics is a computational technique widely used to evaluate protein stability over time. The energy analysis of a protein conformation ensemble allows us to understand the relationship between the protein dynamics and protein mechanism of action.

HINT is a LogP_o/w_-base energy scoring used as a sensitive and rapid tool to evaluate protein intramolecular stability and characterize interaction-pattern changes in determining conformational transitions.

In this work, the HINT-based analysis of Molecular Dynamics trajectories was applied to elucidate the PPARγ mechanism of activation and understand the synergic co-binding effects in glitazones–fatty acids complexes.

PPARγ is a nuclear receptor that regulates the expression of genes involved in glucose and lipid metabolism, as well as in angiogenesis, carcinogenesis, and anti-inflammatory processes.

This nuclear receptor presents a large and flexible active site with a characteristic Y-shape, divided into two sub-pockets known as orthosteric and alternate sites. The flexibility of the ω-loop delimiting the active site has been related to the PPARγ ability to bind more than one ligand simultaneously.

To elucidate the molecular basis behind its complex mechanism of activation, we focused on the PPARγ structure with Rosiglitazone and both Rosiglitazone and Oleic Acid.

Molecular Dynamics simulations revealed the stability of both analyzed systems, with significantly reduced ω-loop flexibility in the PPARγ–Rosiglitazone–Oleic Acid complex.

The HINT-based analysis of produced MD trajectories played a key role in characterizing the system’s intramolecular stability, suggesting that a second ligand bound to the alternate site significantly stabilizes the region between the ω-loop and the helix H3. In particular, the detailed analysis of the HintTable output files unveiled different hydrogen bond contributions to the total intramolecular energy score and mapped all intramolecular interactions occurring between atom pairs. This method allows for an accurate and comprehensive investigation of all protein conformation states and an energy evaluation at the molecular level.

## Figures and Tables

**Table 1:** PPARγ active site characteristics: The active site analysis was carried out in F-pocket. PPARγ active site presents a higher volume than other nuclear receptors and a prevalent hydrophobic character (hydrophobic score is almost triple polar).

Parameters	Score
Volume	1413.471
Total SASA	261.437
Polar SASA	96.962
Apolar SASA	164.475
Hydrophobicity score	48.681
Polarity score	17
Charge score	2
Flexibility score	0.431

**Table 2:** HINT profiles. The average total HINT score and the average total hydrogen bond, electrostatic, and hydrophobic contributions were calculated for both system (PPARγ–Rosiglitazone and PPARγ–Rosiglitazone–Oleic Acid) and all produced trajectories (Run1, Run2, and Run3). Protein systems present a comparable total HINT score but differ for the hydrogen bond energy contribution to the intramolecular stability, suggesting a different intramolecular interaction pathway.

System	Total HINT Score (Average)	Hydrogen Bond (Average)	Electrostatic (Average)	Hydrophobic (Average)
PPARγ–Rosiglitazone (Run1)	5.02 × 10^4^ ±2.36 × 10^3^	5.27 × 10^4^ ±1.53 × 10^3^	3.01 × 10^4^ ±2.11 × 10^3^	2.74 × 10^4^ ±9.59 × 10^2^
PPARγ–Rosiglitazone (Run2)	4.96 × 10^4^ ±2.33 × 10^3^	5.12 × 10^4^ ±1.92 × 10^3^	3.14 × 10^4^ ±2.04 × 10^3^	2.68 × 10^4^ ±5.73 × 10^2^
PPARγ–Rosiglitazone (Run3)	4.94 × 10^4^ ±2.44 × 10^3^	5.27 × 10^4^ ±1.59 × 10^3^	2.99 × 10^4^ ±2.20 × 10^3^	2.71 × 10^4^ ±5.09 × 10^2^
PPARγ–Rosiglitazone–Oleic Acid (Run1)	5.03 × 10^4^ ±1.83 × 10^3^	5.37 × 10^4^ ±1.30 × 10^3^	2.91 × 10^4^ ±1.73 × 10^3^	2.64 × 10^4^ ±5.49 × 10^2^
PPARγ–Rosiglitazone–Oleic Acid (Run2)	5.05 × 10^4^ ±2.01 × 10^3^	5.43 × 10^4^ ±1.54 × 10^3^	3.02 × 10^4^ ±1.89 × 10^3^	2.59 × 10^4^ ±5.28 × 10^2^
PPARγ–Rosiglitazone–Oleic Acid (Run3)	5.08 × 10^4^ ±2.12 × 10^3^	5.46 × 10^4^ ±1.56 × 10^3^	3.06 × 10^4^ ±1.93 × 10^3^	2.74 × 10^4^ ±5.07 × 10^2^

## Data Availability

All structural data were extracted from the Protein Data Bank. All algorithms and formulas for calculations that we performed are presented within this manuscript and/or in the references within. Readers with questions or who wish to access HINT are encouraged to contact the corresponding authors.

## Supplementary Materials

The following supporting information can be downloaded at https://www.mdpi.com/article/10.3390/molecules29102234/s1, Table S1: PPARγ and Estrogen Receptor binding pocket; Table S2: Protein RMSD values; Figure S1: Ligand RMSD profile; Table S3: Ligand RMSD values; Figure S2: HINT score profiles; Figure S3: HintTable output file.
