# Supplementary material for: A Combined Molecular Dynamics and Hydropathic INTeraction (HINT) Approach to Investigate Protein Flexibility: The PPARγ Case Study
Source: Molecules. 2024 May 10;29(10):2234. doi: 10.3390/molecules29102234 (PMC11124508; doi:10.3390/molecules29102234)
Supplement: Supplementary file 1 [file molecules-29-02234-s001.zip › molecules-2988500-supplementary.pdf]

Table of Contents

**Table S1.** PPARγ vs Estrogen Receptor Binding pocket	S1
**Table S2**. Protein RMSD values	S2
**Figure S1**. Ligand RMSD profile	S3
**Table S3**. Ligand RMSD values	S4
**Figure S2.** HINTscore profiles	S5
**Figure S3.** HintTable output files	S6

Table S1 PPARγ (PDB ID: 1FM6) active site characteristics compared to Estrogen Receptor alpha (ERα) (PDB ID: 2YJA). The analysis was carried out in Fpocket using the default parameters settings and reveals a significant PPARγ active site higher volume and flexibility

Parameters	Score (PPARγ)	Score (ERα)
Volume	1413.471	493.171
Total SASA	261.473	98.965
Polar SASA	96.962	62.499
Apolar SASA	164.475	36.466
Hydrophobicity Score	48.681	33.960
Polarity Score	17	5
Charge Score	2	1
Flexibility Score	0.431	0.150

Table S2 Protein RMSD values. The average protein backbone RMSD values for the PPARγ – Rosiglitazone and the PPARγ – Rosiglitazone – Oleic Acid complexes show a high stability of both system over time in all three produced trajectories

	PPARγ - Rosiglitazone	PPARγ – Rosiglitazone – Oleic Acid
RMSD Protein Cα Run1	2,60 ± 0,12	2,14 ± 0,20
RMSD Protein Cα Run2	2,24 ± 0,17	1,74 ± 0,17
RMSD Protein Cα Run3	2,20 ± 0,31	1,62 ± 0,16

Figure S1 Ligand RMSD profile. The stability of Rosiglitazone and Oleic Acid was evaluated by computing the RMSD values during the simulation time for all three independent replicas (run1 in blue, run2 in orange and run3 in grey) Both ligands remain stable during the simulation time thanks to the intensive interaction network with residues in the orthosteric and alternate sites. Figure S1A shows the Rosiglitazone RMSD profile in the PPARγ – Rosiglitazone complex, while Figure S1B and Figure S1C the Rosiglitazone and the Oleic Acid RMSD profile in the PPARγ – Rosiglitazone – Oleic Acid complex.

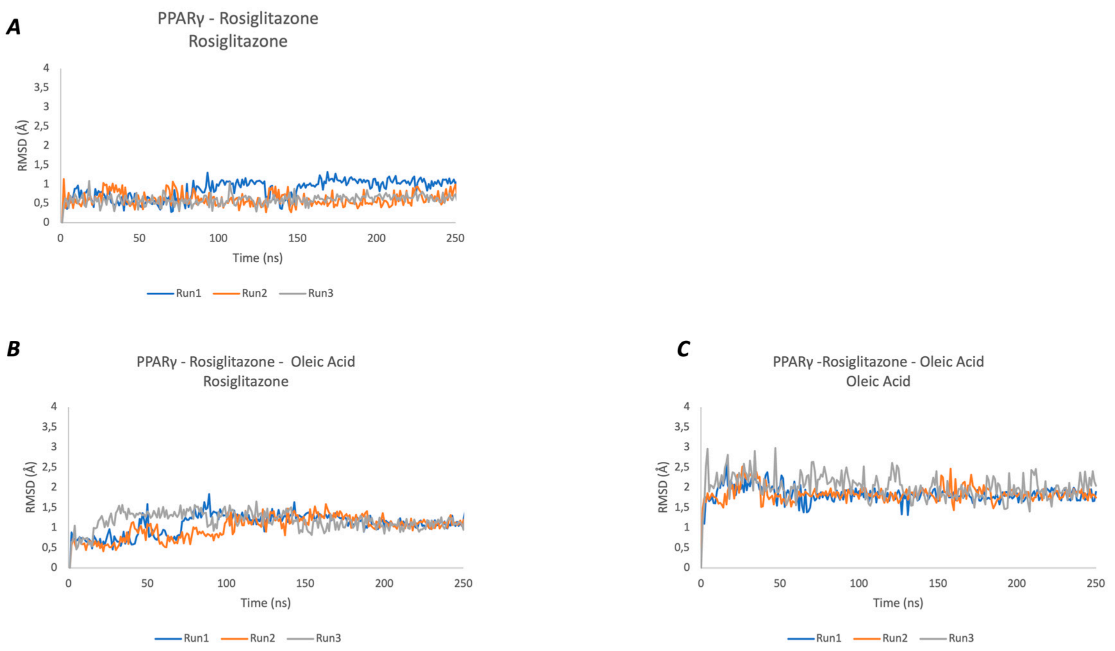

Table S3 Ligand RMSD values. The average ligand heavy atoms RMSD values for the PPARγ – Rosiglitazone and the PPARγ – Rosiglitazone – Oleic Acid complexes show a high stability of both ligand over time in all three produced trajectories

	PPARγ - Rosiglitazone	PPARγ – Rosiglitazone – Oleic Acid
Rosiglitazone Run1	0,86 ± 0,25	1,08 ± 0,26
Rosiglitazone Run2	0,61 ± 0,17	1,01 ± 0,27
Rosiglitazone Run3	0,60 ± 0,12	1,17 ± 0,23
Oleic Acid Run1		1,83 ± 0,22
Oleic Acid Run2		1,84 ± 0,22
Oleic Acid Run3		2,02 ± 0,32

Figure S2 HINTscore values for for the PPARγ – Rosiglitazone (A) and the PPARγ – Rosiglitazone – Oleic Acid (B) complexes over time in all three produced trajectories. The total HINT score and Hydrogen bond, Electrostatic and Hydrophobic contributions are represented as green, orange, grey and yellow lines, respectively

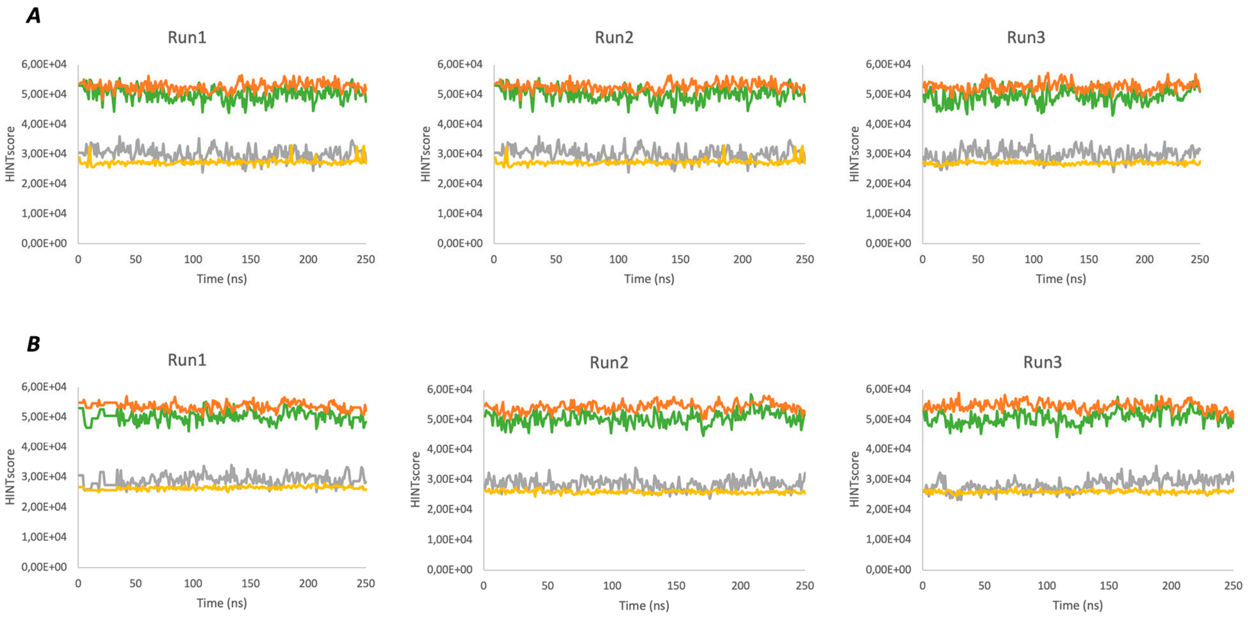

Figure S3 HintTable output file. The HINT output file is a table that lists and describes all intramolecular interactions occurring by interacting atom pairs. The analysis of the output files (PPARγ-Rosiglitazone complex in A and PPARγ-Rosiglitazone-Oleic Acid in B) underlines a different intramolecular interactions pattern involving ω-loop residues. The Oleic Acid binding to the alternate site triggers a protein conformation change that involves both ω-loop and helix H3 residues with a significant stabilization effect.

A PPARγ–Rosiglitazone System

Monomer Type	Monomer Name	Atom Name	Atom Type	Hydrophobic Atom Const.	SASA	Monomer Type	Monomer Name	Atom Name	Atom Type	Hydrophobic Atom Const.	SASA	Distance Angstroms	Distance VDW	Interaction Score	Interaction Type
ILE	267	CB	C.3:1	0.220	6.00	THR	268	N	N.am	−0.224	32.00	3.709	114.14	6	Hydroph./Polar
THR	268	O	O.2	−1.915	26.00	LEU	270	N	N.am	−0.703	32.00	3.497	114.67	34	Hydrogen Bond
PRO	269	C	C.co	1.642	1.00	LEU	270	CB	C.3:2	0.520	23.00	3.579	105.26	3	Hydrophobic
PRO	269	C	C.co	1.642	1.00	LEU	270	CD2	C.3:3	0.820	65.00	4.002	117.69	3	Hydrophobic
LEU	270	N	N.am	−0.703	32.00	GLN	271	CA	C.3	0.190	12.00	4.292	132.07	12	Hydroph./Polar
GLU	272	OE1	O.co2	−3.310	36.00	SER	274	N	N.am	−0.224	32.00	3.666	120.21	114	Hydrogen Bond
GLU	272	OE2	O.co2	−3.310	36.00	GLN	273	N	N.am	−0.519	32.00	3.853	126.31	62	Hydrogen Bond
GLU	272	N	N.am	−0.519	31.00	ARG	280	NH2	N.pl3	−1.540	49.00	5.307	171.18	−10	Acid/Acid
GLU	272	N	N.am	−0.519	31.00	GLN	273	N	N.am	−0.519	32.00	2.862	92.32	−19	Acid/Acid
LYS	275	N	N.am	−0.334	32.00	GLU	276	N	N.am	−0.519	31.00	2.794	90.14	−31	Acid/Acid
GLU	276	OE1	O.co2	−3.310	36.00	ARG	357	NH1	N.pl3	−1.540	49.00	5.045	165.40	13	Acid/Base
GLU	276	OE1	O.co2	−3.310	36.00	ARG	357	NH2	N.pl3	−1.540	49.00	3.042	99.73	264	Hydrogen Bond
GLU	276	OE2	O.co2	−3.310	36.00	ALA	278	N	N.am	−0.703	32.00	2.993	98.15	265	Hydrogen Bond
GLU	276	OE2	O.co2	−3.310	36.00	ALA	278	CB	C.3:3	0.810	75.00	3.403	106.35	−163	Hydroph./Polar
PHE	282	CB	C.3:2	0.489	29.00	ILE	456	CD1	C.3:3	0.820	74.00	5.594	164.54	4	Hydrophobic
PHE	282	CD1	C.ar	0.355	19.00	CYS	285	CB	C.3:1	0.489	30.00	5.061	148.87	3	Hydrophobic
PHE	282	CD1	C.ar	0.355	19.00	LEU	452	CD2	C.3:3	0.820	65.00	4.520	132.94	3	Hydrophobic
PHE	282	CE1	C.ar	0.355	36.00	GLN	286	CG	C.3:2	0.500	35.00	4.451	130.90	2	Hydrophobic
PHE	282	CE1	C.ar	0.355	36.00	LEU	452	CD2	C.3:3	0.820	65.00	4.218	124.06	6	Hydrophobic
PHE	282	CE1	C.ar	0.355	36.00	LEU	453	CD2	C.3:3	0.820	65.00	4.881	143.55	3	Hydrophobic
PHE	282	CZ	C.ar	0.355	40.00	GLN	286	CG	C.3:2	0.500	35.00	4.578	134.65	2	Hydrophobic

B PPARγ–Rosiglitazone–Oleic Acid System

Monomer Type	Monomer Name	Atom Name	Atom Type	Hydrophobic Atom Const.	SASA	Monomer Type	Monomer Name	Atom Name	Atom Type	Hydrophobic Atom Const.	SASA	Distance Angstroms	Distance VDW	Interaction Score	Interaction Type
LYS	265	OX2	O.co2	−3.310	45.00	ILE	267	N	N.am	−0.703	31.00	3.376	110.68	80	Hydrogen Bond
LYS	265	OX2	O.co2	−3.310	45.00	THR	268	N	N.am	−0.224	32.00	4.243	139.12	27	Acid/Base
ILE	267	CA	C.3	0.220	9.00	ILE	281	CD1	C.3:3	0.820	74.00	4.025	118.38	6	Hydrophobic
ILE	267	CG2	C.3:3	0.820	53.00	THR	268	CG2	C.3:3	0.810	67.00	4.436	130.48	29	Hydrophobic
PRO	269	CD	C.3:1	0.588	30.00	GLU	272	CG	C.3:2	0.500	38.00	4.812	141.52	3	Hydrophobic
PRO	269	CD	C.3:1	0.588	30.00	GLN	273	CA	C.3	0.190	12.00	5.074	149.23	3	Hydrophobic
LEU	270	N	N.am	−0.703	32.00	GLN	271	CA	C.3	0.190	12.00	4.292	132.07	3	Hydroph./Polar
GLU	272	OE1	O.co2	−3.310	36.00	GLN	283	NE2	N.pl3	−1.540	49.00	2.773	90.91	344	Hydrogen Bond
GLU	272	O	O.2	−1.915	23.00	SER	274	N	N.am	−0.224	32.00	3.174	104.05	45	Hydrogen Bond
GLU	272	O	O.2	−1.915	23.00	LYS	275	N	N.am	−0.334	32.00	3.209	105.22	95	Hydrogen Bond
LYS	275	NZ	N.4:3	−2.214	61.00	GLN	283	OE1	O.2	−1.915	40.00	3.018	98.95	405	Hydrogen Bond
LYS	275	NZ	N.4:3	−2.214	61.00	ASP	462	OD2	O.co2	−3.310	36.50	5.161	169.20	64	Acid/Base
LYS	275	NZ	N.4:3	−2.214	61.00	ASP	462	O	O.2	−1.915	25.00	2.799	91.76	290	Acid/Base
GLU	276	O	O.2	−1.915	23.00	ILE	279	N	N.am	−0.703	31.00	3.281	107.58	60	Hydrogen Bond
GLU	276	O	O.2	−1.915	23.00	ARG	280	N	N.am	−0.297	32.00	2.818	92.39	122	Hydrogen Bond
PHE	282	CZ	C.ar	0.355	40.00	LEU	356	CD1	C.3:3	0.820	66.00	4.030	118.53	13	Hydrophobic
PHE	282	CZ	C.ar	0.355	40.00	LEU	356	CD2	C.3:3	0.820	65.00	4.282	125.94	9	Hydrophobic
PHE	282	CZ	C.ar	0.355	40.00	ILE	456	CD1	C.3:3	0.820	74.00	3.853	113.34	9	Hydrophobic
PHE	282	CE2	C.ar	0.355	38.00	LEU	356	CD2	C.3:3	0.820	65.00	4.938	145.25	15	Hydrophobic
PHE	282	CE2	C.ar	0.355	38.00	ILE	456	CG2	C.3:3	0.820	53.00	4.042	118.88	6	Hydrophobic
PHE	282	CE2	C.ar	0.355	38.00	ILE	456	CG1	C.3:2	0.520	27.00	4.283	125.97	3	Hydrophobic
PHE	282	CE2	C.ar	0.355	38.00	ILE	456	CD1	C.3:3	0.820	74.00	3.768	110.82	9	Hydrophobic
PHE	282	CD2	C.ar	0.355	18.00	ILE	456	CG2	C.3:3	0.820	53.00	3.401	100.03	11	Hydrophobic
PHE	282	CD2	C.ar	0.355	18.00	ILE	456	CD1	C.3:3	0.820	74.00	3.974	116.87	5	Hydrophobic
ILE	281	CG2	C.3:3	0.820	53.00	PHE	282	CB	C.3:2	0.489	29.00	5.496	161.65	3	Hydrophobic
ILE	281	CG2	C.3:3	0.820	53.00	PHE	282	CE2	C.ar	0.355	38.00	4.872	143.30	3	Hydrophobic
ILE	281	CG2	C.3:3	0.820	53.00	PHE	282	CD2	C.ar	0.355	18.00	4.492	132.12	4	Hydrophobic
